# PROTEIN AI Advisor: A Knowledge-Based Recommendation Framework Using Expert-Validated Meals for Healthy Diets

**DOI:** 10.3390/nu14204435

**Published:** 2022-10-21

**Authors:** Kiriakos Stefanidis, Dorothea Tsatsou, Dimitrios Konstantinidis, Lazaros Gymnopoulos, Petros Daras, Saskia Wilson-Barnes, Kathryn Hart, Véronique Cornelissen, Elise Decorte, Elena Lalama, Andreas Pfeiffer, Maria Hassapidou, Ioannis Pagkalos, Anagnostis Argiriou, Konstantinos Rouskas, Stelios Hadjidimitriou, Vasileios Charisis, Sofia Balula Dias, José Alves Diniz, Gonçalo Telo, Hugo Silva, Alex Bensenousi, Kosmas Dimitropoulos

**Affiliations:** 1 Information Technologies Institute, Centre for Research and Technology Hellas, GR 57001 Thessaloniki, Greece; 2 School of Biosciences and Medicine, Faculty of Health and Medical Sciences, University of Surrey, Guildford GU2 7XH, UK; 3 Department of Rehabilitation Sciences, Katholieke Universiteit Leuven, 3001 Leuven, Belgium; 4 Department of Endocrinology, Diabetes and Nutrition, Charité, 10117 Berlin, Germany; 5 Department of Nutritional Sciences and Dietetics, International Hellenic University, GR 57400 Thessaloniki, Greece; 6 Institute of Applied Biosciences, Centre for Research and Technology Hellas, GR 57001 Thessaloniki, Greece; 7 Department of Electrical and Computer Engineering, Aristotle University of Thessaloniki, GR 54124 Thessaloniki, Greece; 8 Centro Interdisciplinar de Estudo da Performance Humana (CIPER), Universidade de Lisboa, Estrada da Costa, Dafundo, 1499-002 Lisbon, Portugal; 9 PLUX, Wireless Biosignals, 1050-059 Lisbon, Portugal; 10 Intrasoft International SA, 1253 Luxembourg, Luxembourg

**Keywords:** personalized nutrition, meal plan recommendations, artificial intelligence, ontology

## Abstract

AI-based software applications for personalized nutrition have recently gained increasing attention to help users follow a healthy lifestyle. In this paper, we present a knowledge-based recommendation framework that exploits an explicit dataset of expert-validated meals to offer highly accurate diet plans spanning across ten user groups of both healthy subjects and participants with health conditions. The proposed advisor is built on a novel architecture that includes (a) a qualitative layer for verifying ingredient appropriateness, and (b) a quantitative layer for synthesizing meal plans. The first layer is implemented as an expert system for fuzzy inference relying on an ontology of rules acquired by experts in Nutrition, while the second layer as an optimization method for generating daily meal plans based on target nutrient values and ranges. The system’s effectiveness is evaluated through extensive experiments for establishing meal and meal plan appropriateness, meal variety, as well as system capacity for recommending meal plans. Evaluations involved synthetic data, including the generation of 3000 virtual user profiles and their weekly meal plans. Results reveal a high precision and recall for recommending appropriate ingredients in most user categories, while the meal plan generator achieved a total recommendation accuracy of 92% for all nutrient recommendations.

## 1. Introduction

Food is of utmost importance to human physical and mental health as it not only provides the necessary energy for the functioning of the human body, but is also a vehicle that promotes joy, friendship, and social intercourse. However, not all types of food are equally beneficial to overall health, as their component nutrients are processed differently by the human body. According to the World Health Organization (WHO), non-communicable diseases, such as cardiovascular diseases and diabetes, are responsible for 71% of all deaths worldwide yet are preventable through effective interventions that tackle shared risk factors, such as unhealthy diets [1].

As a result, maintaining a well-balanced and nutritious diet is crucial for human health. However, food globalization, lifestyle changes, economic, and socio-cultural factors significantly challenge people’s ability to adhere to healthy diets [2]. Therefore, food intake monitoring systems can provide substantial benefits to users by suggesting ways to adapt to and maintain a healthy diet. However, to keep users engaged with a healthy diet and to achieve optimal results and benefits, more personalized approaches to nutrition support may be required. Personalized nutrition has been formally defined as nutritional advice, tailored to suit an individual based on their personal health status, lifestyle, nutrient intake or alternatively on genetic and phenotypic data [3]. To this end, several authors have highlighted the necessity to design and develop food intake monitoring systems that can work in tandem with nutritional experts to provide optimized personalized healthy diets that are adapted to user-specific parameters. Such parameters might include physical characteristics, dietary choices, health conditions, and preferences, as well as user behaviours (e.g., consider potential deviations from the prescribed diet) and measurements (e.g., glucose levels from IoT devices) in real-time [4,5,6].

This work, which has been carried out within the framework of an ongoing EU-funded project, namely “PROTEIN- PeRsOnalized nutriTion for hEalthy living” [7,8], aims to provide a novel artificially intelligent (AI) personalized nutritional advisor that can generate daily and weekly meal plans after taking into consideration: (a) user profiles (e.g., physical characteristics, dietary choices, health conditions, preferences, etc.), (b) nutrition experts’ recommendations regarding macro- and micronutrients intake, and c) a database of meal plans developed by nutrition experts for varying population groups. Through a series of fuzzy rules and filtering mechanisms, the proposed nutritional AI advisor selects suitable meals from a pool of available meals defined by experts. To achieve this, meals are filtered based on explicit user preferences (i.e., user-defined favorites) and health conditions (e.g., gluten-free products for users with allergies, low-fat meals for users with excess weight/obesity, etc.). Afterwards, the suitable meals are combined to form 24-h meal plans, effectively leveraging AI to achieve personalized nutrition tailored to users’ preferences and needs.

The main contributions of this work are summarized as follows:

- We propose a knowledge-based recommendation framework adopting a novel 2-layered architecture that includes (a) a qualitative layer for verifying ingredient appropriateness, implemented as an ontology-based decision support system acting on the meal level, and (b) a quantitative layer acting as a diet plan generator that optimizes meal plans within expert-defined ranges and thresholds regarding users’ daily consumption. The proposed architecture disentangles the appropriateness of meals from that of meal plans and allows the independent evaluation of each subsystem.
- We introduce a dataset of expert-validated meals along with their respective meal plans, i.e., meal plans created and reviewed by experts in Nutrition. We argue that such a dataset ensures appropriateness in recommendations, which is essential for any health-related recommender system. We make our dataset available at https://doi.org/10.5281/zenodo.7143234 (accessed on 20 October 2022).
- We present thorough evaluations of the proposed framework, combining four key elements that an ideal meal plan recommender should abide to, namely, meal and meal plan appropriateness, meal variety, and finally, system capacity for recommending meal plans. We present results from three types of experiments: (a) a small-scale experiment (200 meals) for verifying the appropriateness of the recommended meals, (b) a large-scale experiment (3000 virtual users and 21,000 meal plans) for evaluating the advisor’s meal plan accuracy and variability, and (c) a medium-scale experiment (300 virtual users) for examining the system’s recommendation capacity as affected by user profile complexity.

The remainder of the paper is organized as follows. Section 2 provides a background of recommender systems and previous works in food recommendation. Section 3 presents an overview of the proposed AI-based nutritional advisor, along with a detailed description of its main components, while Section 4 describes the experiments and presents the results that validate the subsystems of the proposed framework. Finally, Section 5 concludes the paper by discussing the benefits of the proposed nutritional advisor for users that want to maintain a well-balanced and healthy diet.

## 2. Related Work

With the significant impact of nutrition on non-communicable diseases, there is a great need for food recommendation systems capable of shifting users towards healthy and sustainable diets. Generally, recommender systems are the ones producing individualized recommendations or guiding users in a personalized way towards interesting or useful choices from a large pool of possible options [9]. The interested reader may find comparative evaluations and reviews of health recommendation systems and food recommendation systems in the works of De Croon et al., Theodoridis et al., and Trattner and Elsweiler [10,11,12]. Food recommender systems are often categorized based on the method used to recommend healthy diets, namely content-based (CB), collaborative filtering-based (CF) and hybrid approaches. Moreover, recommendations can take the form of individual foods [13] or meals [14], but also more complex items like recipes [15], or even 24-h meal plans [16,17]. A detailed analysis concerning the types of food recommender systems can be found in [18].

Content-based approaches rely on users’ individual tastes, activities and profile to tailor recommendations. In [15,19], recipe recommendations were made based on the collection of scores on a 5-point Likert scale from users who rated their preferences for specific foods or recipes. In a later work, Harvey et al. proposed a food recommendation system to accurately estimate the preference of a user for a specific recipe based on the analysis of the user’s ratings for a set of recipes and their contents (e.g., ingredients, healthiness, etc.) [20]. On the other hand, Teng et al. proposed two networks to generate recipe recommendations by utilizing ingredient complements and substitutes based on regional user preferences [21]. Complement networks of ingredients are constructed via co-occurrence of the same ingredients in the same recipes, while substitute networks are derived from user-generated suggestions for modifications. Experiments show that such an approach significantly outperforms methods that rely on features, such as ingredient list, cooking method, style, etc. Finally, an Augmented Reality (AR) system was proposed in [22] to read the barcode of products at a grocery shop and offer recommendations on healthier alternative products based on user preferences, as well as a prediction for the impact of these products on users’ health. A disadvantage of content-based approaches is the cold-start problem, meaning that they cannot offer appropriate food recommendations to new users that have not yet declared their preferences. Finally, the evaluation of such systems is based on explicit user feedback which, in most cases, is hard to collect [23].

Collaborative filtering-based approaches attempt to find similarities between user profiles and, as a result, make recommendations that would be appropriate for similar users. In [19], the authors tested a nearest neighbour approach using Pearson correlation on the ratings matrix and found out that it offered poorer performance than their proposed content-based approach. On the other hand, the authors in [20] showed that Singular Value Decomposition (SVD) outperformed both the content and collaborative filtering approaches suggested by Freyne et al. [19]. The authors in [24] developed a mobile application with a food recommendation system that relies on matrix factorization to fuse ratings and users’ supplied tags and managed to achieve significantly better prediction accuracy than content-based and standard matrix factorization baselines. In [25], the authors tested various collaborative filtering-based approaches using a large dataset crawled from the online recipe portal allrecipes.com [26] and found that the highest performing CF approaches were Latent Dirichlet Allocation (LDA) [27] and Weighted matrix factorization (WRMF) [28]. Recently, an efficient recommendation system that utilizes a medical dataset to automatically detect which food product should be given to which patient, was proposed [29]. Detection was based on patient’s disease states and other features, such as age, gender, weight, calories, protein, fat, sodium, fibre, and cholesterol. Similarly, the authors in [30] proposed a recommendation system to identify what kind of food product a patient with special needs should consume. Their system was based on patients’ disease and other factors such as weight, gender and age using Blockchain technologies for enhanced privacy and security of the sensitive personal data. A huge disadvantage of the CF approaches is the fact that they rely only on healthy recommendations without considering users’ preferences, and thus can be ineffective as users can easily lose their motivation to adapt and maintain the recommended diet.

Hybrid approaches combine CB and CF to leverage the advantages of each technique and avoid their drawbacks. Such a hybrid approach was initially proposed by Trang et al. [31], deducing that developing food recommendation systems that balance user preferences and nutritional needs is the optimal strategy to follow. Although healthy food and ‘tasty food’ are not mutually exclusive, there is often an optimization trade-off between nutrient intake and user preferences [32]. To achieve this, most hybrid approaches rely on either pre-filtering or post-filtering, depending on whether nutritional needs or user preferences are filtered first [33].

Pre-filtering approaches usually involve constraint-based recommender systems, which are relatively rare in the food domain [32]. Yang et al. proposed a food recommendation system that allows a user to disclose dietary constraints (e.g., halal, vegetarian, or vegan), as well as the user’s nutritional expectations concerning calories, protein, and fat (increase, decrease or maintain), leading to an initial selection of meals [14]. Then, user preferences were employed to re-rank the set of meals and recommend the optimal one. The authors finally recruited users to evaluate their system and provide qualitative feedback. Ribeiro et al. proposed a meal recommender that first estimates the nutritional requirements of the user from user-provided information, such as age, sex, weight, height, and activity level (using a Fitbit activity tracker) and then recommends food items based on criteria, such as a user’s dietary choices, avoidance of recipe repetition within the same week and promotion of meat for lunch and fish for dinner [34]. The proposed meal recommender could produce daily meal plans and was validated through user questionnaires. In a similar fashion, a multi-criteria decision analysis to filter out foods that do not meet a user’s health requirements, before considering the user’s overall preferences, was proposed in [35]. In this work, the proposed recommender produces personalized daily meal plans that were evaluated through the optimization of a traditional diet planning scheme proposed by Anderson and Earle [36].

On the other hand, post-filtering approaches retrieve a relevant set of meals based on user preferences, after which a nutrient-based re-ranking of meals is conducted. Elsweiler et al. developed a food recommendation system that retrieves all recipes with a score above a certain user preference threshold and re-ranks them based on one or more health indicators [17]. Then, the food recommendation system uses the top recipes to propose daily meal plans. Similarly, a post-filtering approach was proposed in [12] that generates balanced daily meal plans by initially filtering meals based on user ratings and then evaluating the healthiness of the meals based on an aggregate health indicator. This indicator takes into consideration the WHO and the Food Standards Agency (FSA) nutritional recommendations for the specific user. Wayman and Madhvanath provide automated, personalized, and goal-driven dietary guidance to users based on grocery receipt data [37]. User preferences were initially identified by the grocery receipts, and then the proposed approach provided recommendations so that the users achieve the recommended dietary reference intake values found in nutrition databases.

Contrary to the previous works, Starke et al. studied how the ranking of optimal meals affects users’ selection of a meal option and how users can be motivated to select healthy meal options by re-ranking the optimal meals based on personalized goals [38]. The evaluation of the proposed approach was performed by employing 17 users that selected a single meal from a choice of 4 meals. In another work [39], the same authors studied how the inclusion of food images (i.e., visual attractiveness) alongside meals can shift users towards healthier meal options, evaluating their approach by employing 239 users who stated their preferred meal out of a set of options. Finally, in [40], the authors proposed a food recommendation system that takes into consideration users’ ratings of ingredients to predict preferred meals in a set of meal options. Afterwards, meal options were re-ranked based on a Cholesterol Factor and the top meals were retrieved. The Cholesterol Factor tracks down recipes’ nutrient content and penalizes those with high levels of cholesterol, while rewarding those with relatively low levels of fat, saturated fat, and sugar. Different meal selection algorithms were evaluated with relevance to user preferences and the score of the Cholesterol Factor of the retrieved meals.

In this work, an AI-based nutritional advisor is proposed that can be categorized as a knowledge-based recommender system for meal plans. The proposed nutritional advisor initially takes into consideration user information such as age, sex, weight, height, dietary choices, health conditions and preferences and identifies a list of appropriate meals that satisfy the personalized nutritional needs of the specific user through a powerful ontology of nutritional rules [41]. This was defined by experts in nutrition and physiology and is in accordance with the European Food Safety Authority (EFSA) recommendations on nutrition. Then, daily meal plans are generated through an optimization process and are provided as recommendations to the user. As a result, the proposed AI-based nutritional advisor overcomes the drawbacks of CB and CF approaches by considering both nutritional needs and user preferences, while it employs an ontology of rules to impose nutritional constraints on the users, thus guiding them towards healthy diets.

## 3. Methodology

**Figure 1:**
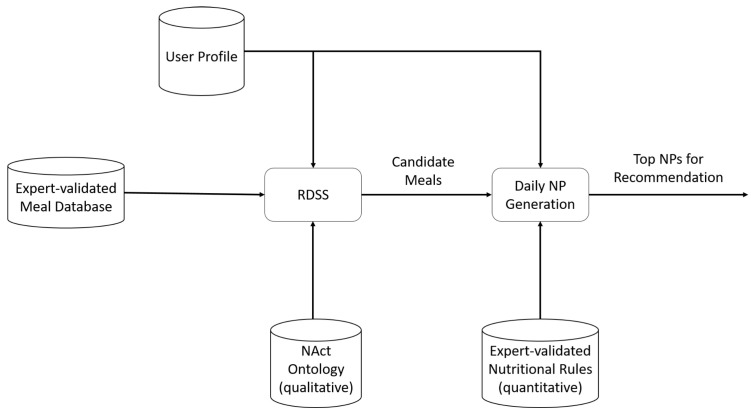
Architecture of the AI-based nutritional advisor.

The proposed nutritional advisor can provide daily nutritional plans (NPs) to its users through the interpretation of their respective profiles. More specifically, the advisor consists of two components, the Reasoning-based Decision Support System (RDSS) and the *NP* generation component. The RDSS generates the set of appropriate meals for a user based on user profile information, the set of available meals, and an ontology of qualitative rules acquired from Nutrition experts [41]. The *NP* generation component combines the appropriate meals to form daily meal plans for recommendation. By adopting such a decoupled architecture, we can reason better about the system’s recommendation accuracy since we can evaluate the recommended meal plans in terms of both meal and meal plan appropriateness, as well as meal variety among meal plans, all independently of each other. These components, along with their interconnections, are presented in Figure 1 and described in detail below.

### 3.1. Supported User Groups and User Profile Modelling

Our system targets 10 user groups including healthy adolescents, seniors and adults, as well as adults with various health conditions and athletes (Table 1). These user groups can be further grouped into three main categories:

- Healthy people that would not be expected to require any specialist supervision.
- People that would be expected to require nutrition specialist supervision.
- People with health conditions that would be expected to require nutrition and medical specialist supervision.

Within the PROTEIN project, nutritional and medical experts modelled these groups, producing distinct user profile models for the three supergroups (A, B, and C). The profile models consist of a list of profile variables that capture key physical characteristics, dietary choices, health conditions, and preferences of the user, as well as accompanying reference ranges, priority, and other attributes of these variables. For simplicity reasons in the present experimental study, we use a unified profile model for all user groups. The unified user profile model captures key physical characteristics of the user (e.g., sex, age, etc.), that are needed for dietary calculations, as well as the dietary preferences (e.g., vegan, halal etc.) and health conditions (i.e., intolerances, deficiencies, allergies, and medical conditions) of the user. A detailed view of the profile model variables is provided in Table 2.

### 3.2. NAct Ontology

One of PROTEIN project’s objectives was to develop an ontology, modelling evidence-based expert knowledge. Previously developed AI expert systems suggested the alteration of one variable of an individual’s lifestyle, per each consideration of any meal plan adjustment. Whereas, the PROTEIN knowledge-based expert system methodology, the background basis of which is the ontology, adopts a holistic approach, involving semantic entities and rules. Through this, we were able to connect (all) user’s implicit and explicit nutritional and well-being goals, and these goals with the situational condition of the subject and standardized European nutritional and well-being directives.

Therefore, the Nutrition and Activity (NAct) ontology [41] was developed as an integral part of the PROTEIN project, serving as the reference knowledge base of the Reasoning-based Decision Support System (RDSS) component of the AI advisor. Previous research yielded several key European and International food and nutrient databases complete with few pre-existing nutritional ontologies, which do not account for rules or relationships between foods/nutrients and user circumstances (diets and/or conditions). Of the few ontologies that were identified within the literature, most lacked rich semantic correlations, presented naive information or lacked the key components that were required for PROTEIN’s AI advisor.

The two most relevant ontologies found are the Food Ontology (FOKB) [44] and the HeLiS ontology by Dragoni et al. [45]. Both model food types and nutritional information about them, with the FOKB delving into details about properties of food products, including additives and governing agents (e.g., anticaking, antifoaming) and HeLiS modelling foods and nutrients as well as physical activities. Both these ontologies manifest the shortcomings identified above, therefore verifying the need to create a new expert-consolidated ontology that covers all the requirements and knowledge of the PROTEIN project.

**Figure 2:**
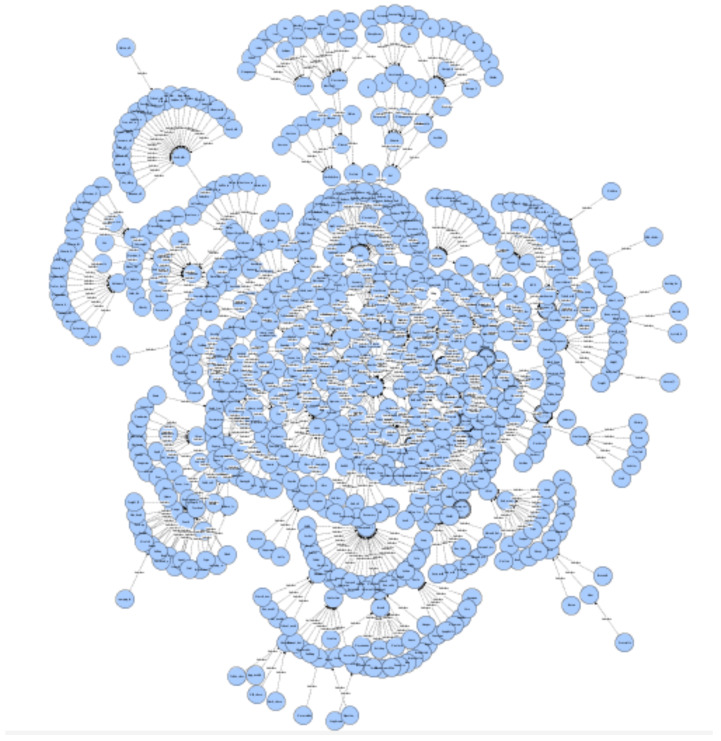
Birds-eye view of the NAct Ontology, v1.9.5, with over 750 interconnected classes.

NAct ontology was constructed based on the Methontology methodology [46], following the methodology’s seven stages of development: specification, knowledge acquisition, conceptualisation, integration, implementation, evaluation, and documentation. Apart from specification, which was meticulously defined early in the ontology’s lifecycle, all other stages followed several iterations of close collaboration and co-creation sessions among health and nutrition experts and the technical team (ontology engineers). Several experts-engineers workshops, trials, and evaluation sessions of the implemented versions of the ontological model, as well as a piloting cycle with user feedback, were conducted in this process, that has led to the current version v1.9.5 of NAct ontology (Available on GitHub at: https://github.com/nutritionactivityontology/nact) (accessed on 20 October 2022). A birds-eye view of the over 750 ontology classes and their interconnections can be seen in Figure 2.

The major axes of the NAct ontology consist of (but are not restricted to) what is presented in Table 3.

### 3.3. Reasoning-Based Decision Support System

The RDSS is a knowledge-based expert system that performs complex decision-making and inferencing through logical reasoning over experts’ knowledge, user profiles and the available meal options stored in a database to achieve a first-level filtering of meals and provide a list of candidate meal options to the *NP* generation component. Therefore, in its core, the RDSS comprises a powerful fuzzy inference engine, namely the Lightweight Fuzzy Description Logic Reasoner (LiFR) [47].

The RDSS allows for semantic matchmaking of candidate meal options against user information, all under the prism of a particular semantic vocabulary and background knowledge. To this end, the RDSS considers:

- NAct ontology. Comprises an amalgamation of European guidelines relevant to nutrition and well-being. It also includes experts’ knowledge, pertaining to foods and their relation to nutrients and their well-being consequences, restrictions and specific nutritional needs relevant to user conditions, as described in the previous section.
- User profile. Each user profile consists of pre-declared, explicit information (user conditions, diet, goals).
- Available meals. A database of over 2000 consolidated meals, created by nutritional experts and used by the RDSS to reason upon and subsequently filter in terms of suitability to the user.

The RDSS is responsible for the pre-filtering of meals to:

- Reject meals which contain foods that are incompatible with a patient’s profile (e.g., would trigger one or more of their allergies or are restricted by doctors due to their medical condition).
- Promote meals and/or restaurant menu items which contain foods that in turn contain nutrients that the user needs to consume more of based on their profile. For instance, this might be due to an explicit goal, or a medical condition induced goal, to increase the intake of a certain nutrient.

The advantages of the RDSS are that it excludes or promotes certain meal constituents that are unwanted or even potentially hazardous to the user. It can also boost constituents as per the user’s health needs and thus alleviates the cold-start problem that occurs whenever new meals or new users are introduced to the proposed nutritional advisor. The latter is achieved by leveraging the nutritional information of each meal and its suitability according to the user group and the explicit preferences declared by the user. The output of the RDSS is a list of acceptable meal options that are then fed to the *NP* generation component for the formation of nutritional plans.

### 3.4. Nutritional Plans Generation

The *NP* generation component is responsible for creating daily nutritional plans based on the quantitative rules for nutrients created by a group of experts, such as medical experts, nutritionists, as well as, physical activity experts. The generation of each plan also depends upon the list of appropriate meals recommended by the RDSS. More specifically, the *NP* generation component receives as input:

- Expert validated nutritional rules: These are quantitative nutritional rules validated by the group of experts, that define the target energy intake (calories per day), as well as appropriate ranges of macronutrients (i.e., carbohydrates (CHO), fat, saturated fatty acids (SFA) and protein) and other dietary components (i.e., fibre and fruits and vegetables). A meal plan should abide by those rules, based on the user’s profile and the personalized goals selected by the user (i.e., increase or lose weight). The experts classified these ranges as essential (i.e., the *NP* must abide by them), desirable (i.e., the *NP* should abide by them) and non-essential (i.e., the *NP* can disregard them).
- User profile: Information about gender, user groups and medical conditions are important to define which nutritional rules should be applied to the users.
- Candidate meals: A list of appropriate-for-the-user meals recommended by the RDSS.

#### 3.4.1. Expert Validated Nutritional Rules

Experts in the fields of nutrition and dietetics worked towards proposing specific recommendations on macronutrient and micronutrient reference intakes and intakes of other important dietary components. To this end, the experts utilized the most up-to-date evidence published by EFSA and WHO to shape their recommendations [48,49,50] as illustrated in Table 4 and Table 5 below (EI, Energy Intake; BW, body weight; 1 portion is equivalent to 80 g. Dark background denotes that the corresponding reference intake values are essential for this user group, an intermediate background denotes that the corresponding reference intake values are desirable, while a white background denotes that the corresponding reference intake values are non-essential.) for two example user groups, adults with obesity and with Type 2 diabetes respectively.

The rules are used by the *NP* generation component that utilizes the list of candidate meals to fill daily meal plans by randomly placing and combining meal options for the different meals of the day (i.e., breakfast, morning snack, lunch, afternoon snack, dinner, and supper). To evaluate the appropriateness of each generated *NP* for a given user profile, a fitness function is defined that evaluates how much a NP fits the specific user profile. In this work, the fitness function is defined as follows:

(1) $$NP_{fitness}=S_{calories}\times S_{macronutrients}\times S_{micronutrients},$$

In the above equation, $S_{calories}$ is the absolute difference between the energy intake of the recommended *NP* (i.e., sum of the calories of all meals in a day) and the target energy intake defined by the nutritional experts for this type of user. Moreover, $S_{macronutrients}$ and $S_{micronutrients}$ are defined in a similar way using the following equation:

(2) $$S_{*}=\prod_{i=1}^{N_{*}}w_{i}p_{i},$$

where * stands for either macronutrients or micronutrients, *N* is the number of macronutrients or micronutrients, $w_{i}$ is a weight factor that is equal to 1 if the specified nutrient is classified by experts as essential or desirable, otherwise $w_{i}$ is equal to 0 and $p_{i}$ is a penalty, defined as follows:

$$p_{i}=\left\{\begin{array}{cc} ‘PenaltyValue^{’}, & \mathrm{if}\;\mathrm{nutrient}\;\mathrm{falls}\;\mathrm{out}\;\mathrm{of}\;\mathrm{KT}\;\mathrm{range}\\ ‘AwardValue^{’}, & \mathrm{if}\;\mathrm{nutrient}\;\mathrm{falls}\;\mathrm{within}\;\mathrm{KT}\;\mathrm{range}\\ ‘EssentialAwardValue^{’}, & \mathrm{if}\;\mathrm{nutrient}\;\mathrm{falls}\;\mathrm{within}\;\mathrm{KT}\;\mathrm{range}\;\mathrm{and}\;\mathrm{is}\;\mathrm{essential} \end{array}\right.$$

Similar to [34], our modelling scheme involves scores which are defined either as distances from thresholds or award/punishment values for ranges defined in KT. Extra awards are given to NPs that fall into the red-highlighted ranges of KT, e.g., iron for ‘Adults with Iron Deficiency’ or CHO for ‘Adults with Type 2 Diabetes’. In this context, we have also defined a fixed value of $10,10^{-1}$, and $10^{-2}$ for penalty, award, and essential award values, respectively. These values have been determined through a trial-and-error process.

Meal variety is also a central aspect of our recommendation framework. Establishing variety in the results of a recommender system poses a challenge since it is directly related to the attractiveness of the system but also has a negative effect on performance, as described in [23]. To address these shortcomings, we have defined several variety filters, described in the following.

**Filtering out repeating foods.** A *NP* is composed of meals, and each meal may contain any number of foods (i.e., ingredients). We aim to reduce the number of repeating foods within each *NP*, and specifically to limit each food to one appearance per *NP*, to exclude repeating recommendations that might be frustrating to users. For example, users do not want to see ‘bananas’ being recommended as part of both morning snack and dinner. Regarding the technical details of implementing this type of filter, our logic awards an extra value to the *NP* if it does not contain the same food twice. Otherwise, the number of repeating foods is used as a weight in the calculation of the overall *NP* fitness (i.e., the greater it is, the larger the final distance and the less optimal the *NP* is).

**Filtering out repeating meals.** We also consider the case in which different NPs might contain the same meals. In such cases, we want to reduce the number of repeating meals recommended every week. Therefore, we defined a threshold with which a certain meal can be contained in up to three different NPs. Technically, the filtering of repeating meals takes place after all NPs have received a fitness score, so that we can start iterating from the fittest solution and proceed in decreasing order.

**Filtering out repeating meal sequences.** Apart from individual meal repetitions, there is also a chance that the same sequence of meals might be repeating in different NPs within each week. For that reason, we have defined a threshold with which a certain sequence of meals cannot be contained in any two NPs. Like the previous filter, repeating meal sequences are filtered after fitness evaluation is completed.

#### 3.4.2. PROTEIN Meal Database

A total number of 381 24-h meal plans accounting for all user groups were created. As shown in Table 6, the 24-h meal plans consist of 3 separate meals (breakfast, lunch, and dinner) and 3 snacks (mid-morning, mid-afternoon, and evening), as shown in Table 6. The plans were devised utilising healthy eating principles such as incorporating the recommended number of fruit and vegetable portions in accordance with EFSA [52] and WHO recommendations [53]. Furthermore, for specific user groups such as adults with cardiovascular disease (CVD) and type 2 diabetes mellitus (T2DM), wholegrains have also been identified as an important component of the diet and were incorporated within the 3 separate meals created. Meal plans developed for other specific user groups, such as adults with iron deficiency anaemia, were similarly tailored, in this case with an emphasis on including varied iron sources as well as optimising vitamin C, which has been shown to enhance the absorption of iron [54]. As the PROTEIN Project is an EU trial, cultural dietary choices were also carefully considered. An example was the incorporation of common foods found within the Mediterranean diet to provide meal plans for participants from Greek and Portuguese institutions. It was also important to ensure that there were foods provided on these meal plans that were accessible to individuals from different countries. An example is cereal brands and different cheese types (feta for Greek participants/ cheddar for the UK and Edam for Germany/ Belgium), to optimise user engagement and satisfaction.

To create the required meal plans, we first created a meal plan name according to the naming convention of ‘partner name–group code–calories–index–gender–diet’. As an example, a meal plan devised for a female, vegetarian user with iron deficiency, created by the University of Surrey experts, would be entered into the system as: UoS-C1a-1500-001-F-Vegetarian. Gender was presented as either M, F or A (male, female, or all) while diet was specified as NA (not applicable), Vegetarian, VegetarianL (lacto), VegetarianO (ovo), Vegan, Pescatarian, RMA (red meat avoider), Halal or Kosher.

Following this, we used the Nutrium dashboard [55] and its food composition data and ingredient lists to ‘build’ the proposed meals and snacks. Moreover, country-specific databases were used where available, for example, the UK meal plans were populated exclusively using the McCance and Widdowson food tables [56]. Furthermore, experts chose in their raw state or the most simplified cooked option, e.g., chicken would be listed as the raw cut that the partner wants the user to specifically utilise (such as the chicken breast/thigh/wing) for the meal. Following this, the cooking method was also specified, and the food quantity was amended, as required, and listed as g/portion. Where specific foods could not be found within the Nutrium database of foods, we added custom foods.

Following initial meal plan creation, it was agreed that these templates should be supplemented with recipes/instructions on how to combine and cook the foods listed, especially for some of the lunches and dinners where more than basic preparation was required. The list of different cooking methods and serving suggestions currently available for use in the recipes are shown below in Table 7. Table 8 illustrates the creation of a recipe by selecting appropriate instructions for each ingredient.

## 4. Experimental Results

For the evaluation of the proposed recommendation framework, we considered four main aspects that a real-time nutritional advisor should retain: (a) meal appropriateness, (b) system capacity for generating meal plans (c) meal plan appropriateness, (d) meal variety. The 2-layered, decoupled architecture that was adopted for our suggested knowledge-based recommendation framework allows the evaluation of each of these conditions in isolation, that is, in different experiments. That way, we can get a much more detailed view regarding the recommendation accuracy and capabilities of the system. For each experiment, a number of virtual users were created, and their respective meal plans were generated according to their profile characteristics and user category. Finally, for measuring the performance of the system we employed different evaluation metrics, specific to each experiment.

### 4.1. Recommended Meals’ Appropriateness

Meal rejections due to critical patient conditions consist the most critical aspect of the RDSS. It is the only point in PROTEIN where we make sure to avoid foods and/or nutrients that may pose a health threat to the users. It was thus critical to make sure that no such user-hazardous foods were allowed to pass to the meal plan generation, i.e., that the precision of the RDSS was at 100%. In addition to precision, recall was estimated, to see whether the RDSS was too strict in this process, i.e., rejecting meals that may have been not harmful to the user. Since this is a delicate and critical evaluation, all assessments were done manually, very carefully examining each food in the experiment’s candidate pool. To facilitate this cumbersome process, the candidate pool was reduced to a manageable number of candidates, i.e., 200 meals (out of the total 2266 in the system), randomly selected from all meal categories (breakfasts, snacks, dinners, etc.). The results can be seen in Table 9.

Values depicted are in the [0, 1] interval, effectively corresponding to a 0–100% success rate. The profiles used were also carefully selected to represent simple food restrictions (banana, avocado), direct food restrictions (kosher, banana and avocado, vegan, salicylates, FODMAPs, tree nuts), nutrient restrictions (lactose, gluten, T2D, fructose) and a combination of restrictions (all). In addition, selection sought to cover all kinds of restrictions, i.e., allergies, intolerances, medical conditions, and dietary choices and lastly to cover from less restrictive user profiles (e.g., banana and avocado) to, increasingly, the most restrictive types of user profiles observed in the system (e.g., vegan). The latter (more restrictive user attributes) is directly correlated with the availability of suitable meals in the system that may accommodate such a range of restrictions.

Evidently, the main objective of achieving 100% precision is achieved. In addition, recall is very high, and in some cases 100% accurate, for all different categories, thus proving that the RDSS is not unnecessarily restrictive and accurately takes advantage of the pool of meal candidates in PROTEIN. All recall errors were examined to be due to unnecessary additions in food descriptions that do not affect precision but pose extra restrictions in some cases. e.g., turkey ham was correctly annotated as such, but also as plain ham, which in the ontology is subsumed by pork, thus producing all recall errors for the kosher user.

The RDSS also promotes meals containing ingredients which are high in a particular nutrient, that the user explicitly or implicitly (due to a condition) should increase their intake of. It does not consider the absolute concentration of said nutrient, but rather, based on relevant NAct axioms, it assesses whether foods rich in this nutrient are included in the meal, e.g., promote spinach meals for iron deficiency since spinach is rich in iron. The absolute concentration is afterwards considered in NAP generation component to produce the meal plans. The goal of RDSS at this level is to sort out meals so as to provide an appropriate initial selection for NAP generation component.

It was thus important to assess whether the meals that were promoted were in fact a part of the top-n meals in which the examined nutrient’s concentration is highest. The assessment was made for 4 different values of n and the results can be seen in Table 10. Again, the nutrients-to-increase intake of selection was carefully selected to represent an indicative range of micronutrients and macronutrients, and the pool of candidates is the same randomly selected 200 meals from all categories, as before.

An interesting observation regarding meal promotion by the RDSS is that the results are not improving the more the proposed meals are (i.e., in the top-20), but rather the higher the quality/concentration of the nutrient is in a meal (i.e., being in the top-3). This is most probably because the RDSS promotes meals with foods that are especially rich in the said nutrient, instead of meals that may contain small amounts of different foods that may cumulatively result in the meal being somewhat rich in the said nutrient.

### 4.2. Meal Plan Generation Capacity

Starting from a large but also finite number of validated meals (2266 meals in total) and proceeding with consecutive filtering of the appropriate ones (for each user) by the RDSS and *NP* generation processes, a valid concern is if the AI Advisor can reliably generate meal plans and to what extent, especially for challenging user profiles. To gain insight into these questions, we created up to 300 virtual user profiles and conducted several experiments.

As was detailed in Section 3.1, the user profile contains a multitude of variables to capture the possible dietary choices or health conditions of a user: 9 dietary choices, 8 food intolerances, 12 food deficiencies, 27 allergies, and 16 medical conditions, totalling to 72 conditions. For each variable group the user profile accepts none, or as many as all possible conditions, which leads to a very high number of possible combinations and, thus, possible user profiles (although not all are realistic profiles). To limit the number of possible combinations to a manageable set of virtual user profiles, we set up some rules for profile creation aiming at the creation of both realistic and relatively challenging profiles. Non-realistic profiles are not the focus in the context of this paper, while the obvious assumption is that less challenging profiles would lead to better results.

The rules that have been applied for the creation of realistic virtual user profiles were that: (a) the profile could have up to one condition per group, excluding the medical conditions group, and (b) the profile could have each condition in a group with a probability corresponding roughly to what applies in real life (details are provided below per experiment). The rules that have been applied for the creation of challenging virtual user profiles were that: (c) the profile should have at least one medical condition (and up to two, along with ‘Obesity’), and (d) certain, indicative, most challenging conditions were selected. The latter were identified as the ones that, according to the NAct ontology, rule out the highest number of foods and, thus, meals.

Two experiments were conducted, where up to 300 virtual user profiles were created following the rules and the RDSS and *NP* generator processes were run with a time limit of 2:45 min for the RDSS and 3:15 min for the total processing to simulate near real-time conditions. Two iterations were conducted for each experiment as we consider that, in real life conditions, a second attempt from the side of the real user to create a plan would be probable, in case of a failed first attempt. In the second iteration we processed only those profiles for which the AI Advisor was not able to create a *NP* during the first iteration.

**Figure 3:**
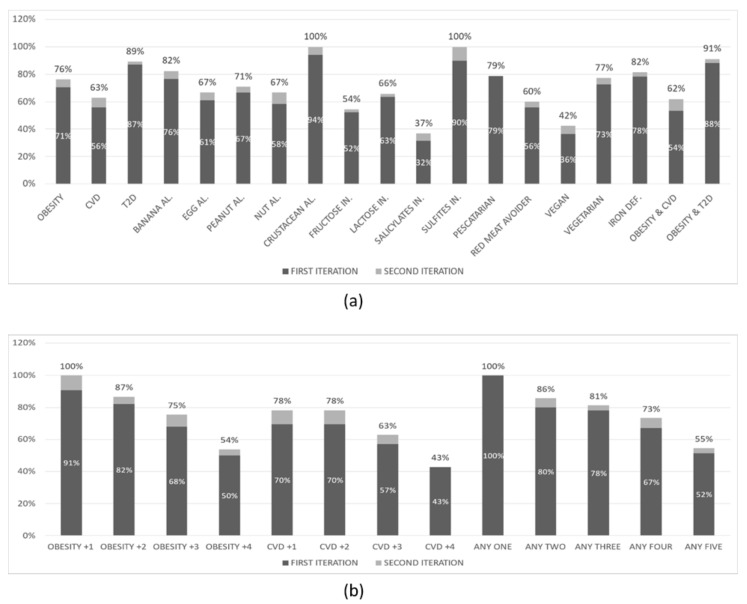
*NP* creation rate for virtual user profiles with (**a**) at least the condition(s) mentioned, and (**b**) exactly the condition(s) mentioned (first experiment).

The results of the first experiment are shown in Figure 3 below. The overall *NP* creation rate was 70% in the first iteration and 75% in the second iteration of this experiment. This experiment has been conducted with 219 virtual user profiles that had:

- None (51%) or one of the following specific food choices: Pescatarian (13%), Red meat avoider (11%), Vegan (15%), Vegetarian (10%)
- None (47%) or one of the following food intolerances: Fructose (21%), Lactose (18.7%), Salicylates (8.7%), Sulphites (4.6%)
- None (70%) or one of the following food deficiencies: Iron (30%)
- None (54%) or one of the following allergies: Banana (8%), Egg (8%), Peanut (11%), Tree nut (11%), Crustacean (8%)
- Heart disease (54%) or Type 2 Diabetes (46%), and Obesity (63%) or not (37%)

**Figure 4:**
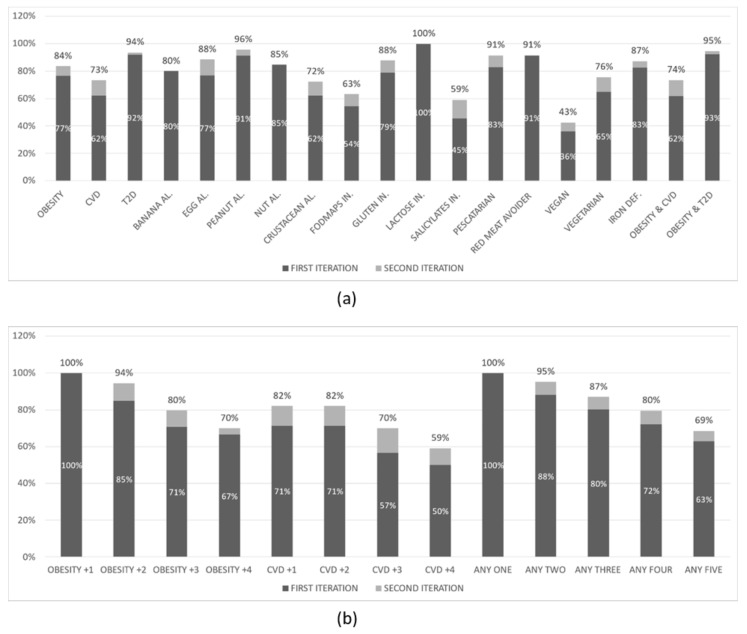
*NP* creation rate for virtual user profiles with (**a**) at least the condition(s) mentioned, and (**b**) exactly the condition(s) mentioned (second experiment).

The results of the second experiment are shown in Figure 4 below. The overall *NP* creation rate was 76% in the first iteration and 83% in the second iteration of this experiment. This experiment has been conducted with 300 virtual user profiles that had:

- None (49%) or one of the following specific food choices: Pescatarian (12%), Red meat avoider (12%), Vegan (16%), Vegetarian (12%)
- None (45%) or one of the following food intolerances: FODMAPs (23%), Gluten (19%), Lactose (6%), Salicylates (7%)
- None (79%) or one of the following food deficiencies: Iron (21%)
- None (53%) or one of the following allergies: Banana (8%), Egg (9%), Peanut (8%), Tree nut (13%), Crustacean (10%)
- Heart disease (53%) or Type 2 Diabetes (47%), and Obesity (65%) or not (35%)

A first observation is that the overall rate in all experiments is ranging from 70% to 83%. From the (b) parts of the figures above we can clearly observe that the *NP* creation rate drops as the number of conditions in the profiles increases. This is expected since an increased number of conditions introduce a progressively increasing number of restrictions on what meals can be included in the suggested dietary plan. In the (a) parts of the figures above we observe that, in most cases, the rate follows approximately the overall rate, ranging from 60% to 100%. There are, however, a few exceptions where the rate drops significantly: when the user profile includes a salicylate (as low as 37%) or fructose (as low as 54%) intolerance or when it indicates a preference for a vegan diet. The low *NP* creation rate in these specific categories of user profiles can be attributed to the relatively small number of appropriate validated meals for such profiles that have been created to date by the PROTEIN project experts.

### 4.3. Meal Plan Accuracy

#### 4.3.1. Experiment Description

The proposed AI-based nutritional advisor is validated through an experiment including 3000 virtual users. The gender, age, height, weight, and health conditions of users were selected based on either normal or uniform distributions to represent a typical population of users for the proposed nutritional advisor. More specifically, the age of users was in the range of 20–65 years old, the gender of users was equally distributed among males and females, the height and weight of males were in the ranges of 1.60–2.00 m and 60–180 kg respectively, while the height and weight of females were in the ranges of 1.50–1.90 m and 40–150 kg, respectively. Based on their profiles, the users were distributed across the 10 user groups presented in Table 11, with more significance given to the CVD, Type-2 Diabetes and Adults with Obesity categories.

#### 4.3.2. Meal Plan Composition

The experiment ran for all 3000 virtual users for a period of 7 days; thus the proposed AI-based nutritional advisor created a weekly meal plan for each user. Each daily meal plan is formulated based on 6 meal types consumed during a day, namely, breakfast, morning snack, lunch, afternoon snack, dinner, and supper. The experiment shows that, for the 21,000 generated daily meal plans (i.e., 3000 users multiplied by 7 days), there are respectively 269 breakfasts, 246 morning snacks, 236 lunches, 231 afternoon snacks, 220 dinners and 48 suppers which are unique, as shown in Table 12. These meal options are selected based on the user profiles (i.e., preferences, user groups, medical conditions, etc.) and the transcoded knowledge of the nutritional experts.

**Figure 5:**
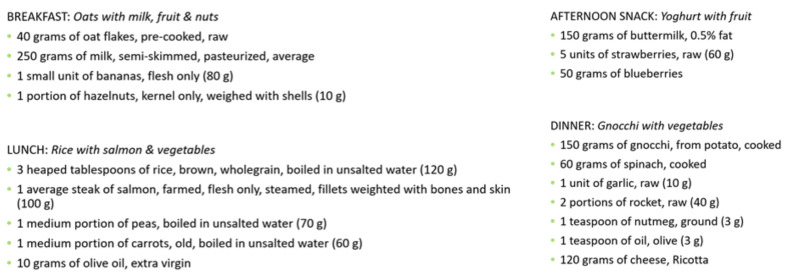
Examples of meal options for breakfast, lunch, afternoon snack and dinner, available in the database of meals.

Examples of meal options available in the database of meals for various meal categories are presented in Figure 5.

#### 4.3.3. Results

Table 13 presents the accuracy of the daily meal plans generated by the proposed AI-based nutritional advisor in terms of macronutrients and other dietary components for the different user groups. A missing value (i.e., “N/A”) is attributed to the fact that the nutritional experts did not provide essential or desirable rules for adults with obesity as far as other dietary components were considered and thus the proposed nutritional advisor is unconstrained to choose the values it sees fit, making the comparison with target values not applicable in this case. The results show that the average macronutrients and other dietary component accuracy between the generated daily meal plans and the target values is 92.65% and 85.86%, respectively, for all user groups. This means that the proposed AI-based nutritional advisor manages to generate appropriate meal plans, whose macronutrients and other dietary components are on average more than 85% probable to be inside the target ranges of the nutritional experts no matter the medical condition of the user. The proposed nutritional advisor achieves the lowest accuracy for the Athletes’ group, which is mainly attributed to the small number of available meal options which meet the recommendations to this specific user group.

As far as energy intake is concerned, a comparison is performed between the values accrued by the generated meal plans of the proposed AI-based nutritional advisor and the personalised daily requirements calculated for each profile. Table 14 presents: the absolute difference in percentage between the recommended and target energy intake values, the Pearson correlation that defines how close the two distributions of the proposed and target energy intake values are, and the results of a t-test analysis (*p*-value).

It can therefore be concluded that the overall mean difference between the proposed and target energy intake is +7.32%, indicating the accuracy of the generated daily meal plans. The largest difference in the energy intake is noticed for Athletes, and it is attributed to the very high energy intake goals (>3000 kcal daily) set for this group, which cannot be easily supported by the existing meal options in the database, thus leading the predicted energy intake values to be usually lower than the required energy intake values.

On the other hand, the metric of Pearson correlation is employed to study the possibility of the proposed energy intake values being close to the target energy intake values by chance. This metric is computed by considering the two sets of energy intake values as distributions and studying their characteristics. From the results, it is shown that for each user group and for all users, the Pearson correlation is above 0.98, meaning that the two distributions are in fact highly correlated with each other. For the overweight adults and especially adults with Type 2 diabetes, the Pearson correlation reaches values above 0.95, which shows an almost perfect correlation. In the same fashion, from the t-test results, it can be deduced that the hypothesis that the two distributions of the proposed and target energy intake values are similar is statistically significant, as shown from the low *p*-values (<0.001). These results demonstrate the ability of the proposed AI-based nutritional advisor to generate daily and weekly meal plans compatible with the plans nutritional experts would recommend for the tested user groups, as far as energy intake is concerned.

**Figure 6:**
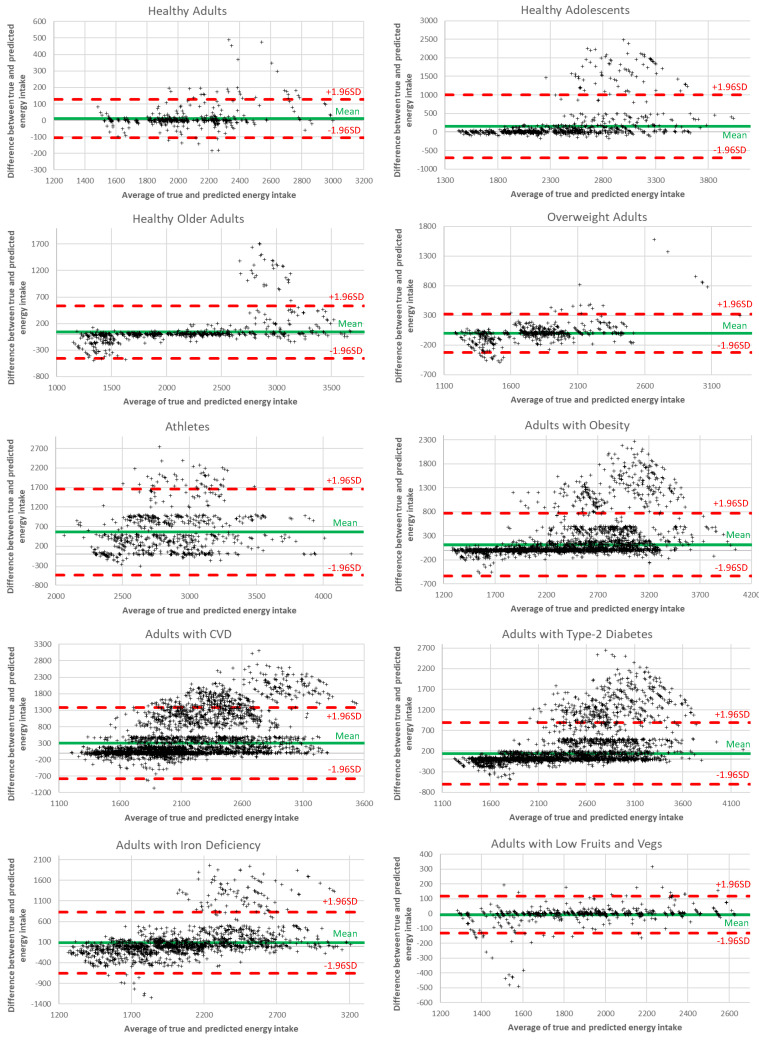
Bland-Altman plots depicting energy intake differences for each user group.

In Figure 6, we analyze the energy intake differences between the ground truth and the recommended values using Bland-Altman diagrams. Each diagram shows the dispersion of the generated meal plans around the mean value, with samples within the red lines (i.e., 1.96 standard deviations from the mean value) considered valid. The results reveal that the percentage of samples falling within the accepted limits surpasses 92% for all user groups, thus revealing the ability of the proposed AI recommendation system to achieve accurate performance.

**Figure 7:**
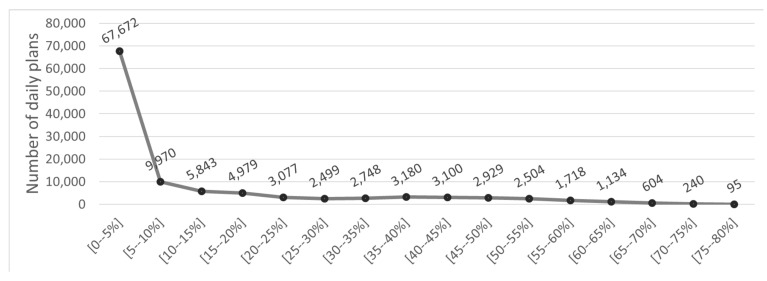
Distribution of energy intake differences between recommended and target values.

In addition, the distribution of the energy intake differences between the recommended and the target values is presented in Figure 7. This figure illustrates that most differences reside in the [0–5%], meaning that most energy intake differences are smaller than 5%. Moreover, the energy intake differences follow a distribution that approximates the normal distribution, with the occurrences of high differences diminishing and only a handful of occurrences of high energy intake differences (>60%). This would be an expected distribution pattern for accurate prediction system.

**Figure 8:**
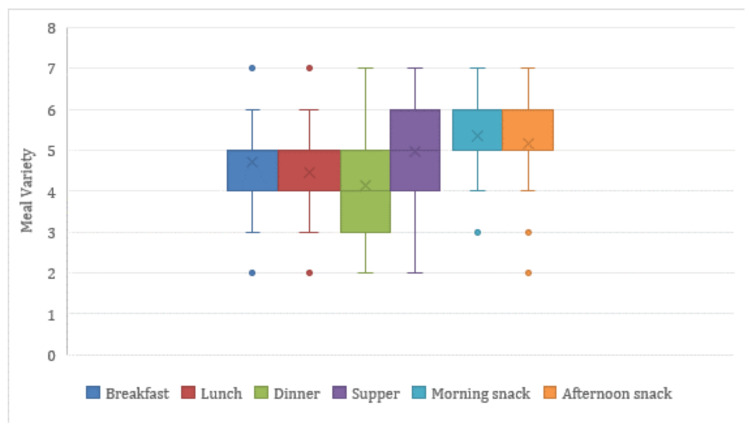
Average meal variety for each meal type in a generated weekly meal plan.

Finally, we performed an extra experiment regarding meal variety for the different meal types of a daily plan, presented in Figure 8. We define meal variability as the number of unique meals proposed for a weekly meal plan generated by the AI-based nutritional advisor. As a result, the meal variety, per meal type, e.g., breakfast, ranges from 1 (i.e., a single meal is repeatedly proposed for each day of a week) to 7 (i.e., a different meal is recommended for each day of a week). A large meal variety is important so that a real user finds the generated weekly meal plan more interesting and appealing.

**Figure 9:**
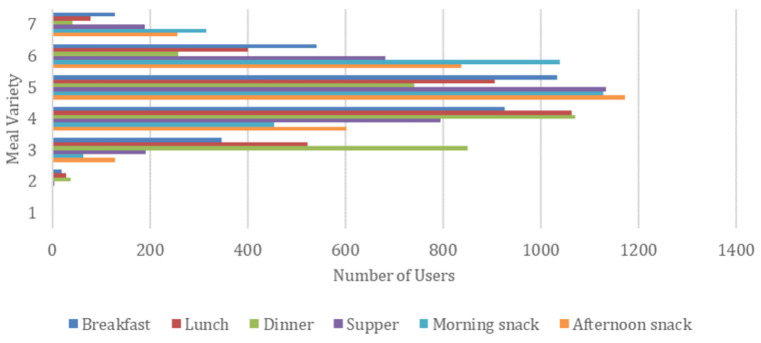
Distribution of meal variety across users.

From Figure 8, it can be deduced that the median meal variety for each meal is above 3.5, meaning that on average the generated weekly meal plans contain 3 or 4 different meals. There are also a few weekly meal plans having every meal different (meal variety equals 7), while the minimum observed meal variety in a plan is 2, as shown in Figure 9. This number is significantly affected by the number of unique meals available in the dataset and the medical condition of a user that eliminates certain meal options. We can also see the differences by meal type, with snacks tended to be the most varied type while dinner being the least varied one. We believe that the average meal variety of 3.5 achieved by the proposed AI-based nutritional advisor is attributed to the filtering techniques aiming to boost variability and allows the generation of weekly meal plans with significant diversity, thus enabling users to better adopt and retain healthy and nutritious diets.

## 5. Discussion and Conclusions

In this paper, we presented a recommendation framework, which adopts a two-stage architecture for modelling diets to provide a safe and appropriate meal plan recommender system. In this context, the PROTEIN AI Advisor utilises evidence-based science from experts in Nutrition and international governing bodies to provide advice for: (i) qualitative rules to avoid potentially harmful ingredients, (ii) quantitative rules to generate daily meal plans for different population groups, and (iii) a database of expert-validated meals. Within this paper we explore the two distinctive parts of the recommendation process and provide an evaluation of the results for meal recommendations from a user-specific perspective, meal plans provided, as well as, the capability of the system to handle and respond to ‘complex’ user profiles.

Our results show that the system was highly accurate at generating appropriate recommendations for macronutrients and other key dietary components within the daily meal plans for all user groups (92.65% and 85.86%, respectively). However, a limiting factor to this accuracy was the number of meals available within the database for the recommender system to draw from. This was shown within the athletes’ group, which had a considerably smaller subset of meals to draw from, contributing to reduced accuracy for the other dietary component recommendations (31.07%). On average, meal variety was predicted to equate to 3–4 meals suggested per meal type over the week, which was considerably lower than anticipated. This could contribute towards lowered user engagement because of the lack of variety. Therefore, in the future it would be expected that the nutritional experts devise further meal plans to be integrated within the system to avoid a lack of meal variety, particularly targeting those user groups with more complex needs.

In addition, the resiliency of the system was verified by exploring the relation between meal plan generation and ‘complex’ user profiles. All restrictions were considered as part of the *NP*, including allergies, intolerances, medical conditions, and dietary choices. We developed a system that should consider an individual from the least (i.e., no allergies/ dietary choices) to the most complex profiles (such as gluten-free, vegan and nut-free). Due to a smaller meal database, the meal variety for complex user profiles was considerably affected. Therefore, the authors acknowledge that the system would benefit from a larger meal database accounting for more complex cases, such as vegans, in the future. However, it was observed that the overall *NP* creation rate was high (between 76–83%) for users with various health conditions, allergies, nutritional deficiencies, and dietary choices, when testing the system on two different occasions. Overall, our study shows a high precision and recall for recommending appropriate ingredients for most user profiles, while the meal plan generator achieved a total recommendation accuracy of 92% for all nutrition recommendations.

Future perspectives for the current framework of the system include the expansion of the architecture with the addition of a third layer of personalization, which requires the evaluation of the system directly from real users in real-time. For instance, the introduction of a star-rating system would be an essential addition that can enable the *NP* to understand an individual’s personal preferences for certain foods and meals. The integration of this third level of personalisation would also pose challenges, since it brings up the need to be evaluated by the research community to verify whether the system has properly interpreted the user preferences in accordance with their nutritional goals and recommendations.

## Figures and Tables

**Table 1:** Supported user groups.

ID	User Group	Description
A1	Healthy Adolescents	Healthy persons between the age of 15 year(s) and 18 year(s).
A2	Healthy Adults	Healthy persons between the age of 18 year(s) and 65 year(s).
A3	Healthy Older Adults	Healthy persons over the age of 65 year(s).
B1	Adults with excess weight	Persons between the age of 18 year(s) and 65 y with a BMI between 25 kg/m ${}^{2}$ and 30 kg/m ${}^{2}$.
B2	Athletes	Persons between the age of 18 year(s) and 65 year(s) with a physical activity level (PAL) of 1.745 and over.
C1	Adults with obesity	Persons between the age of 18 y and 65 year(s) with a BMI of 30 kg/m ${}^{2}$ and over.
C2	Adults with CVD	Persons between the age of 18 year(s) and 65 year(s) with a cardiovascular disease, i.e., a documented disease of the heart and/or blood vessels [42].
C3	Adults with T2D	Persons between the age of 18 year(s) and 65 year(s) with a diagnosis of Type-2 Diabetes from their general practitioner (GP) or hospital consultant. Additionally, adults with a fasting plasma glucose of ≥7 mmol/L (126 mg/dL) or a 2-h plasma glucose of 11 mmol/L (200 mg/dL) during a 75 g oral glucose tolerance test or an HbA1c of ≥48 mmol/mol (≥6.5%). These definitions were created in line with the International Diabetes Federation (IDF) clinical practice guidelines [43].
C4	Adults with Iron Deficiency	Persons between the age of 18 year(s) and 65 year(s) with a diagnosis of iron deficiency anaemia (Haemoglobin < 120 g/L).
C5	Adults with a diet low in fruit and vegetables	Persons between the age of 18 year(s) and 65 year(s) that are consuming less than three portions of fruit and vegetables per day on average.

**Table 2:** A detailed list of all user profile model variables.

User Profile Model Variable Group		Variables of the User Profile Model
Physical characteristics		Age, Sex, Height, Weight, Physical Activity Level (PAL), Body Mass Index (BMI, calculated), Basal metabolic rate (BMR, calculated)
Dietary choices		Pescatarian, Red meat avoider, Vegan, Vegetarian, Lactovegetarian, Lacto-ovo-vegetarian, Ovo-vegetarian, Halal, Kosher, None (i.e., no specific food choice)
Health conditions	Food intolerances	Amines, Caffeine, FODMAPs, Fructose, Gluten, Lactose, Salicylates, Sulfites, None (i.e., no food intolerance)
	Food deficiencies	Calcium, Electrolytes, Folic acid, Iron, Magnesium, Potassium, Sodium, Vitamin A, Vitamin B12, Vitamin C, Vitamin D, Fluid, None (i.e., no food deficiency)
	Allergies	Anise, Avocado, Banana, Celery, Chamomile, Egg, Fish, Garlic, Kiwi, Linseed, Lupin, Milk, Mustard, Passion fruit, Peach, Peanut, Pollen, Sesame, Soy, Strawberry, Sulfite, Sulfur dioxide, Tree nut, Wheat, Gluten, Crustacean, Mollusc, None (i.e., no allergy)
	Medical conditions	Cardiovascular Diseases (CVDs) (Angina, Dyslipidemia, Hypertension, Peripheral artery disease, Previous, Myocardial Infarction, Heart disease), Anemia, Celiac disease, Hypertrophy, Inflammatory Bowel Disease, Kidney disease, Musculoskeletal problem, Obesity, Previous stroke, Type 2 Diabetes, None (i.e., no medical condition)

**Table 3:** Main NAct ontology axes (superclasses/categories).

NAct Axis	Description
**Diet**	Consists of lifestyle-specific choice of diets (e.g., vegan, halal, etc.). Preference-related diets (e.g., Atkins, Mediterranean, etc.) were opted not to be included in the ontology by the experts, since the preference-related options should be learned by the system and not be constricted by such diets. Only critically restrictive lifestyle choice diets were thus included.
**Condition**	Allergies, intolerances, deficiencies and particular medical conditions that may pertain to a patient or the general population, as defined by the user profile model (see previous section) are included in this categorisation.
**Food**	Simple foods (e.g., tomato, pork, orange, etc.) as well as some basic compound foods (e.g., bread, pasta, etc.) that may be used as constituents (ingredients) of meals are included in this category. The most important relations of these foods with conditions and diets are encoded in corresponding rules within the ontology (e.g., a user with banana allergy cannot eat bananas). Also, the breakdown of compound foods to their simple ingredients (based on the basic recipe of each compound food) is encoded in semantic rules (e.g., a biscuit consists of butter, egg, flour, and sugar).
**Nutrient**	All major macronutrients and micronutrients are described in this category. The most important relations of each food to one or more nutrients are encoded in semantic rules within the ontology (e.g., flour is high in gluten, dark leafy greens are high in B9, carotenoids and fibre).
**Property (Goals)**	Several properties of other classes in the ontology are encoded here (e.g., food attributes such as low-fat, wholegrain, etc.), but the most important ones are the goals which a patient (user) wants to explicitly reach with their use of PROTEIN (e.g., increase fibre intake), or that are implicitly mandated by one or more of their medical conditions (e.g. patients with CVD need to reduce salt and fat intake).

**Table 4:** Rules for Adults with Obesity category.

Adults with Obesity	CHO [48] %EI	Protein [50] g/kg (BW)	Fat [49] %EI	Saturated Fat [49] %EI	Fibre [48] g/day(s)	Fruit [51] Portion	Vegetable [51] Portion
Male	45 ± 10%	0.8–1.2	25–30	5–10	30–35	2–5	3–5
Female	45 ± 10%	0.8–1.2	25–30	5–10	20–25	2–5	3–5

**Table 5:** Rules for Adults with Type 2 Diabetes category.

Adults with Obesity	CHO [48] %EI	Protein [50] g/kg (BW)	Fat [49] %EI	Saturated Fat [49] %EI	Fibre [48] g/day(s)	Fruit [51] Portion	Vegetable [51] Portion
Male	45 ± 10%	0.8–1.4	30 ± 10%	5–10	30–45	2–5	3–5
Female	45 ± 10%	0.8–1.4	30 ± 10%	5–10	30–45	2–5	3–5

**Table 6:** Examples of a generic meal plan template and suggested meal titles (suitable for all users).

Meal Plan #	Meal Type	Meal	Meal Title
1	Breakfast	Honey, Yogurt, Milk, Raspberries, Strawberries, Bananas, Cereals	Yogurt with oats, fruit & honey
	Morning snack	Bananas	Banana
	Lunch	Beetroot, Carrots, Cheese, Olive oil, Burger, Quinoa	Beef with quinoa, cheese & fresh vegetables
	Afternoon snack	Strawberries	Strawberries
	Dinner	Turkey slices, Cheese, Pepper, Tortillas, Rocket, Tomatoes, Avocado, Cucumber	Turkey & cheese tortilla with vegetables
2	Breakfast	Mini whole meal toast, Jam and preserves, UHT skimmed milk, Apple	Toast with jam, glass of milk & apple
	Morning snack	Wheat and rye brown bread, eggs, Banana	Brown bread with eggs & banana
	Lunch	Carrot Soup, Wheat and rye bread, Rice, Chicken, Vegetables, Oil, Apple, Orange juice	Carrot soup & chicken and rice with vegetables
	Afternoon snack	Yogurt, muesli, Banana	Muesli & yogurt with banana
	Dinner	Vegetable Soup, Wheat and rye bread, Sweet potato, Roasted pork loin, Vegetables, Oil, Apple	Vegetable soup & roasted pork with sweet potatoes
	Supper	Yogurt, Jam, Nuts	Yogurt with jam & nuts
3	Breakfast	Breakfast cereal, Milk, Smoothies	Breakfast cereal, Milk, Smoothies
	Morning snack	Bananas	Bananas
	Lunch	Sandwich, Potato crisps, Lettuce, Tomatoes	Sandwich, Crisps, Lettuce, Tomatoes
	Afternoon snack	Strawberries	Strawberries
	Dinner	Turkey, Sauce, Spinach, Pepper, Courgette, Creme caramel	Turkey, Spinach, Pepper, Courgette, Dessert
4	Breakfast	Eggs, Bread, Spinach, Peppers	Eggs, Bread, Spinach, Peppers
	Morning snack	Bananas	Bananas
	Lunch	Vegetable stir fry mix, Rice, Chicken, Yogurt	Vegetable stir fry, Rice, Chicken, Yogurt
	Afternoon snack	Salmon, Broccoli, Potatoes, Dressing, Butter	Salmon, Broccoli & Potato
	Dinner	Drinking chocolate powder	Drinking chocolate
5	Breakfast	Muesli, Yogurt	Muesli, Yogurt
	Morning snack	Peaches	Peaches
	Lunch	Rye bread, Radish, Cucumber, Vinegar, Oil, Munster cheese, Yogurt	Open-faced sandwich & salad
	Afternoon snack	Strawberries, Crackers	Strawberries, crackers
	Dinner	Salmon, Potatoes, Soup, Broccoli, Garden peas and carrots	Soup, potato, fish & salmon

**Table 7:** Examples of the meal cooking and preparation options for recipe development within the meal plans.

Amount	Cut	Cook Until	Cut
Weighed (as above)	Whole	Cook through	Simmer in pan
One handful	Cut into cubes	Cook until golden	Stew in saucepan with lid
One cup	Cut into slices	Cook until charred	Casserole in pot with lid
1 tablespoon	Dice finely	Cook until soft	Oven roast
1 teaspoon	Cut in half	Cook al dente	Grill
1 pack	Grate	Cook to preference	Heat through
1/2 pack	Peel into ribbons	**Season/ Mix**	Stir fry
Whole	Cut into wedges	Season with pinch of salt	**Cooking duration**
Halved	Cut into pieces	Season with grind of salt & pepper	5 mins
Quarters	**How to cook**	Season with drizzle of olive oil	15 mins
**Initial Prep**	Follow instructions on packaging	Drain	25 mins
Remove from packaging	Grill (no oil)	Chop & add herbs	35 mins
Remove skin	Grill (lightly oiled)	Sprinkle on herbs	45 mins
Peel Skin	Boiled in unsalted water	Mix in herbs	1 hour
Skin on	Boiled in salted water	Add sauce	1 hour 0 mins
Wash in cold water	Steam	Spread on top	2 hours +
Drain	Poach	Butter or low-fat spread	N/A
Cut stem	Oven roast (lightly oiled)	Add dressing	**Serve**
Rub with oil & seasoning	Fry (no oil)	Drain & mash with butter or seasoning	On a single plate
**Cut**	Fry (lightly oiled pan)	Mix in blender	In a bowl
Whole	Pan cook in butter	**Method**	Eat with hands
Cut into cubes	Bake	Drain	Garnish
Cut into slices	Raw	Boil in unsalted water	Add herbs
Dice finely	Mix	Wash & chop	Drizzle on olive oil
Cut in half	Add to pan	Wash & slice	Add croutons
Grate	Stir fry	Drain & add or heat	Add herbs & oil
Peel into ribbons	Toast	Steam	Add dressing
Cut into wedges	Warm through	Add to pan	Pinch of salt
Cut into pieces	Heat-up	Pour over	Pinch of salt & pepper
**Last Step**	**Combine**	Heat & pour over	Add gherkins
Drain pasta	Mix	Grate on top	Add gherkins & mayo
Drain rice	Pour on top	Peel & slice	N/A
Wash salad	Serve separately	Stir fry	Serve with sour cream
Grate cheese	Stack	Served with	Garnish with mustard
Mix in herbs	Wrap	Fry & add	**Side dish**
Mix salad & dressing	Create sandwich	Bake	Bread & butter
	Add water & stir	Chop	Glass of milk
	Green salad		Potato salad
			Garlic Bread

**Table 8:** Example of the meal cooking and preparation required for a single meal.

**Existing meal title**	Bass, Rice, Pak Choi, Soy Sauce, Cabbage, Carrots, Onions
**New meal title**	Sea Bass & Rice Stir Fry
**(descriptive & user friendly)**	
**Food #1**	Bass, sea, fresh only, baked
**Food #2**	Rice, brown, basmati
**Food #3**	Pak Choi, steamed, whole
**Food #4**	Cabbage, red, raw
**Food #5**	Carrots, old, raw
**Food #6**	Soy Sauce, light & dark varieties
**Method generated**	Bass, sea, fresh only, *baked*, *cook through*
	Rice, brown, basmati, *boiled in unsalted water*, *cook until soft*
	Pak choi, steamed, whole, *stir fry cook al dente*
	Cabbage, red, raw, *stir fry*
	Carrots, old, raw, *stir fry*
	Soy sauce, light & dark varieties, *stir fry*
	*Combine:* mix
	*Serve:* in a bowl

**Table 9:** Evaluation (precision/recall/f-measure) of meal rejections by the RDSS.

Condition/Diet	Precision	Recall	F-Measure
Banana + Avocado allergy	1.000	1.000	1.000
Tree nuts allergy	1.000	1.000	1.000
Salicylates intolerance	1.000	1.000	1.000
Lactose intolerance	1.000	0.995	0.997
FODMAPs intolerance	1.000	0.995	0.997
Gluten intolerance/allergy	1.000	0.990	0.995
Fructose intolerance	1.000	0.960	0.979
T2 Diabetes	1.000	0.990	0.995
Vegan	1.000	1.000	1.000
Kosher	1.000	0.966	0.983
**Average**	**1.000**	**0.989**	**0.995**

**Table 10:** Evaluation (top-n accuracy) of meal promotions by the RDSS.

		Accuracy		
Deficiency/Goal to Increase	Top-20	Top-10	Top-5	Top-3
Protein	1.000	1.000	1.000	1.000
Fat	0.800	0.800	1.000	1.000
Carbohydrates	0.750	0.800	0.800	1.000
Iron	1.000	1.000	1.000	1.000
**Average**	**0.8875**	**0.900**	**0.9500**	**1.000**

**Table 11:** Virtual users’ distribution in PROTEIN’s user categories.

User Group	Number of Users
Healthy adults	85
Healthy adolescents	158
Healthy older adults	131
Overweight adults	92
Athletes	103
Adults with obesity	635
Adults with CVD	706
Adults with T2D	766
Adults with iron deficiency	231
Adults with low fruits & vegs	93

**Table 12:** Unique meal options for the various meals in a daily plan.

Meal Type	Unique Meal Options
Breakfast	269
Lunch	236
Dinner	220
Supper	48
Morning snack	246
Afternoon snack	231

**Table 13:** Recommendation accuracy of the generated daily meal plans in terms of macronutrients and other dietary components.

User Group	Macronutrients Accuracy (%)	Other Dietary Components Accuracy (%)
Healthy adolescents	99.32	89.24
Healthy adults	99.75	97.65
Healthy older adults	99.78	95.09
Adults with excess weight	93.84	94.10
Athletes	82.11	31.07
Adults with obesity	98.66	N/A
Adults with CVD	81.36	N/A
Adults with T2D	97.58	97.24
Adults with iron deficiency	95.55	75.53
Adults with a diet low in fruits and vegetables	99.62	99.66
**Overall accuracy**	**92.65**	**85.86**

**Table 14:** Comparison of energy intake between generated daily meal plans and experts’ recommendations.

User Group	Energy Intake Difference (Mean ± Std)	Pearson Correlation (r)	*p*-Value
Healthy adults	1.18% ± 2.70%	0.999	<0.001
Healthy adolescents	6.84% ± 17.48%	0.985	<0.001
Healthy older adults	4.29% ± 11.28%	0.993	<0.001
Overweight adults	4.49% ± 7.98%	0.995	<0.001
Athletes	18.35% ± 17.57%	0.981	<.001
Adults with obesity	5.40% ± 14.13%	0.990	<0.001
Adults with CVD	14.26% ± 24.48%	0.969	<0.001
Adults with T2D	6.88% ± 16.47%	0.987	<0.001
Adults with iron deficiency	10.15% ± 15.56%	0.984	<0.001
Adults with low fruits & vegs	1.38% ± 3.13%	0.999	<0.001
**Overall**	**7.32% ± 13.08%**	**0.983**	**<0.001**

## Data Availability

The data presented supporting reported results are openly available in Zenodo at https://doi.org/10.5281/zenodo.7143234 (accessed on 14 September 2022).
